# Supplementary material for: Brain-wide single-neuron bases of working memory for sounds in humans
Source: bioRxiv. 2025 Nov 10:2025.11.10.687666. Preprint. [Version 1] doi: 10.1101/2025.11.10.687666 (PMC12642642; doi:10.1101/2025.11.10.687666)

bioRxiv preprint doi: https://doi.org/10.1101/2025.11.10.687666; this version posted November 11, 2025. The copyright holder for this preprint (which was not certified by peer review) is the author/funder, who has granted bioRxiv a license to display the preprint in perpetuity. It is made available under aCC-BY 4.0 International license.

Supplementary Table 1. Proportions of modulated neurons for different task phases.

Proportion of neurons	Early maintenance			Late maintenance			Adjustment		
Region	↑	↓	–	↑	↓	–	↑	↓	–
middle hippocampus	5.8	14.3	79.9	6.5	11.7	81.8	14.3	23.4	62.3
posterior hippocampus	3.4	35.6	60.9	2.3	41.4	56.3	29.9	29.9	40.2
PHG	7.3	15.2	77.5	7.9	15.9	76.2	18.5	25.8	55.6
amygdala	2.4	22.6	75.0	2.9	10.1	87.0	5.8	26.9	67.3
anterior cingulate	3.7	27.8	68.5	4.6	21.3	74.1	21.3	27.8	50.9
middle cingulate	13.7	11.8	74.5	7.8	13.7	78.4	17.6	33.3	49.0
posterior cingulate	19.1	6.4	74.5	0.0	17.0	83.0	42.6	4.3	53.2
anterior insula	4.2	11.1	84.7	1.4	8.3	90.3	30.6	16.7	52.8
posterior insula	9.3	10.9	79.8	13.2	7.8	79.1	41.9	12.4	45.7
gyrus rectus	3.3	18.3	78.3	6.7	15.0	78.3	11.7	23.3	65.0
lentiform nucleus	2.6	21.1	76.3	2.6	17.1	80.3	32.9	14.5	52.6

Supplementary Figure 1. Curation details for all neurons. ISI: inter-spike interval. SNR: signal-to-noise ratio, calculated as the ratio of the peak of each single neuron waveform divided by the median absolute value of the signal for that channel. The bimodal nature of spike waveforms widths as shown in the bottom panel has also previously been observed by others, e.g. [44]

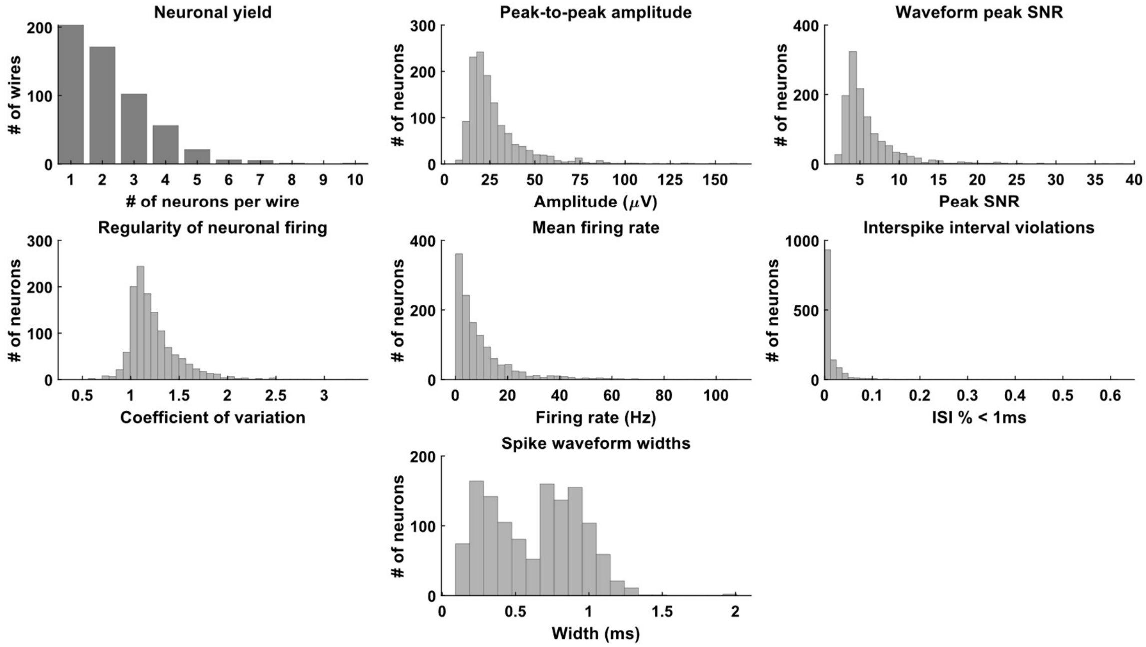

bioRxiv preprint doi: https://doi.org/10.1101/2025.11.10.687666; this version posted November 11, 2025. The copyright holder for this preprint (which was not certified by peer review) is the author/funder, who has granted bioRxiv a license to display the preprint in perpetuity. It is made available under aCC-BY 4.0 International license.

Supplementary Figure 2. Pie chart showing distribution of locations of all single neurons. Number of neurons resolved is shown in parentheses for each location. Naming is shown from top to bottom for the relevant half of the chart. Lentiform nucleus encompasses globus pallidus and putamen. Almost half of all resolved neurons were located within MTL.

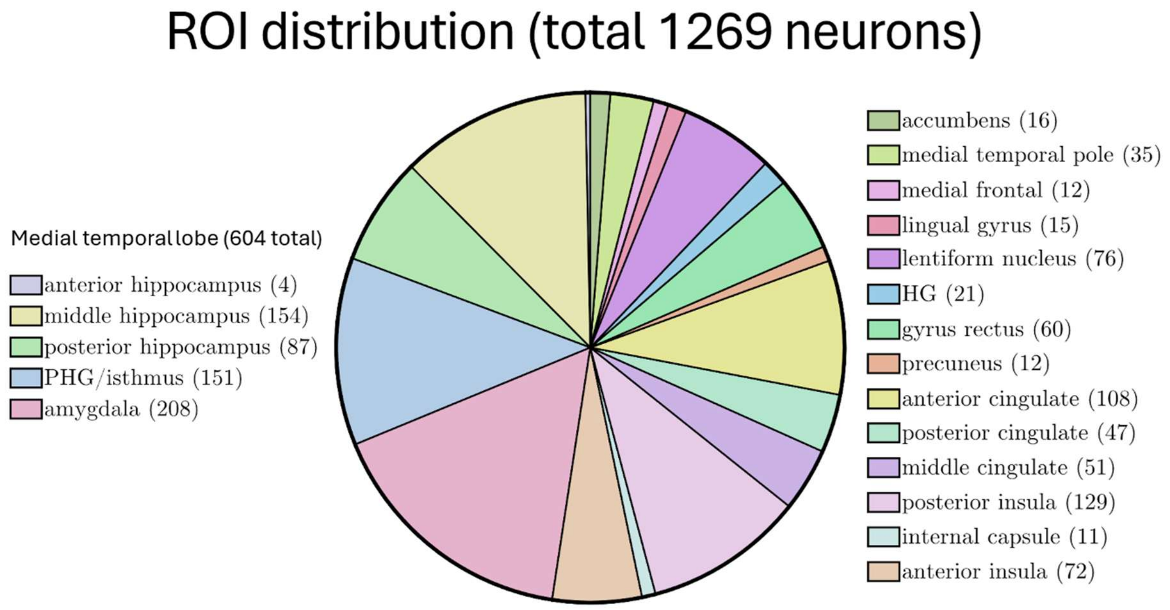

Supplementary Figure 3: Magnitude of changes at task phases of interest. These were calculated for each neuron based on *z*-score relative to baseline (see Methods), then averaged across all isolated neurons within a region. Error bars represent standard error of the mean. Stars indicate significance of deviation from zero, derived from *p*-values based on one-sample t-tests with False Discovery Rate correction applied (* $p< 0.05$, ** $p< 0.01$, *** $p< 0.001$).

bioRxiv preprint doi: https://doi.org/10.1101/2025.11.10.687666; this version posted November 11, 2025. The copyright holder for this preprint (which was not certified by peer review) is the author/funder, who has granted bioRxiv a license to display the preprint in perpetuity. It is made available under aCC-BY 4.0 International license.

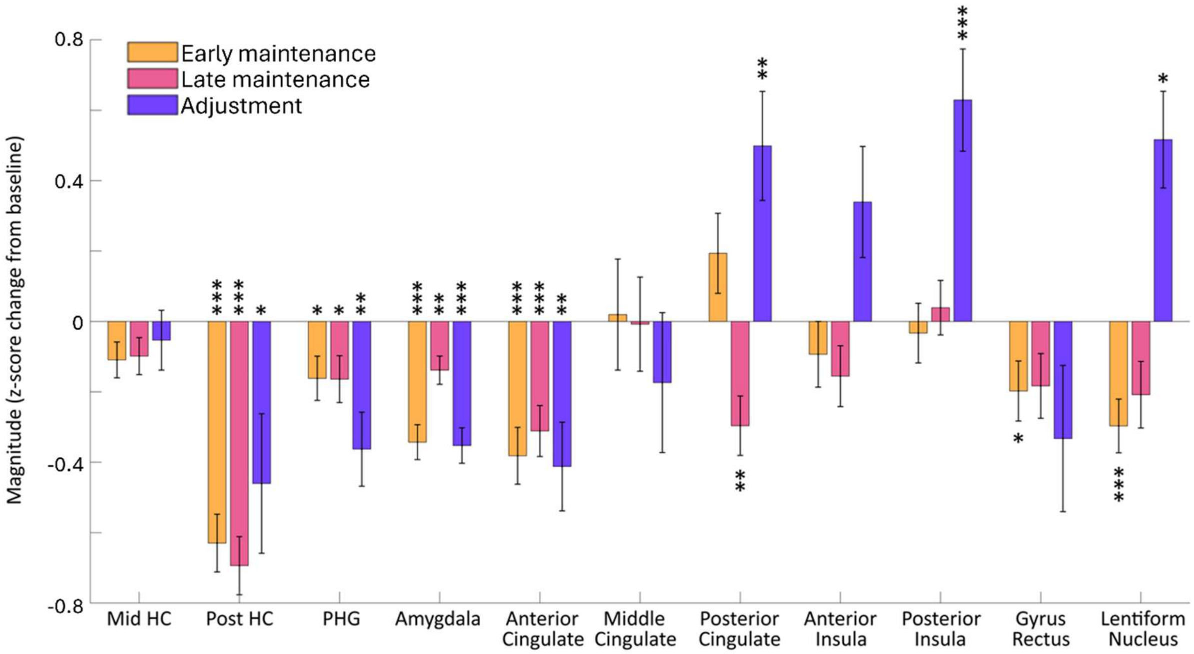

Supplementary Figure 4. Cumulative variance explained as a function of the number of principal components across all isolated neurons.

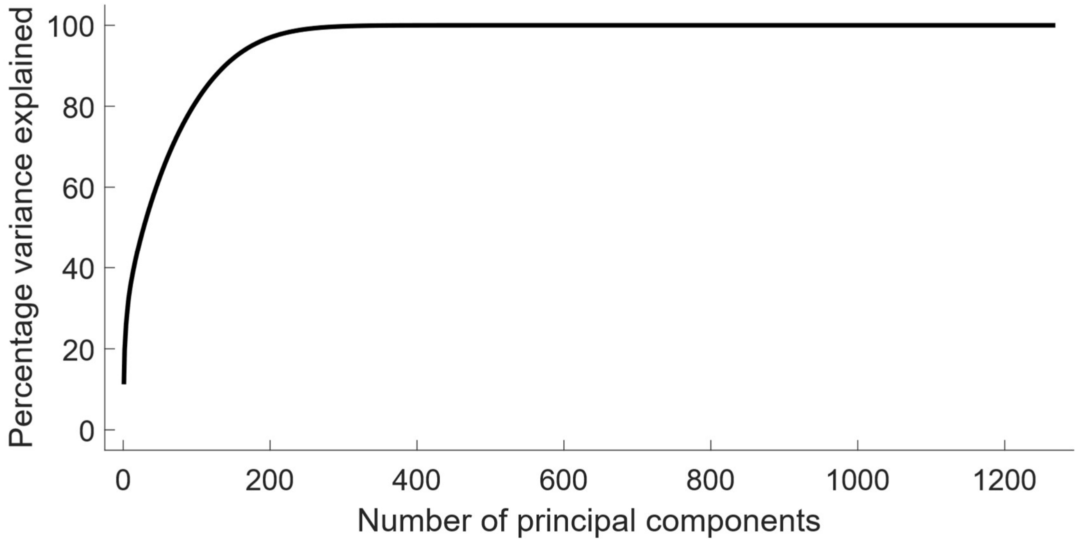

bioRxiv preprint doi: https://doi.org/10.1101/2025.11.10.687666; this version posted November 11, 2025. The copyright holder for this preprint (which was not certified by peer review) is the author/funder, who has granted bioRxiv a license to display the preprint in perpetuity. It is made available under aCC-BY 4.0 International license.

Supplementary Figure 5. Decoding analyses to determine behavioral error across all neurons (upper left panel), tone frequency across all neurons (upper right panel) and tone frequency for Heschl's gyrus (HG) neurons (bottom panel), using a maximum-correlation-coefficient classifier. The latter analysis was mainly included to demonstrate feasibility of the decoding approach, as the sample size was limited for this region (21 neurons in one patient). Periods of significance shown with blue horizontal lines below decoding traces, determined via comparison of true label decoding performance against shuffled trial labels to create a null distribution (shuffled 500 times, alpha value set at 0.002, i.e. 1/500). Horizontal black line indicates chance performance. It is important to note that we constrained the starting tone frequency during adjustment to between 2% and 10% of the target frequency, which highlights that adjustment tones began within a similar frequency range to target. Across all neurons, decoding performance was not significantly above chance at any time during maintenance, for either behavioral error or tone frequency. Behavioral error was significantly above chance for a prolonged window during feedback, suggestive of performance monitoring, and briefly above chance at one 50ms window near the start of adjustment, while tone frequency was only briefly above chance during two separate 50 ms windows during adjustment when considering all neurons.

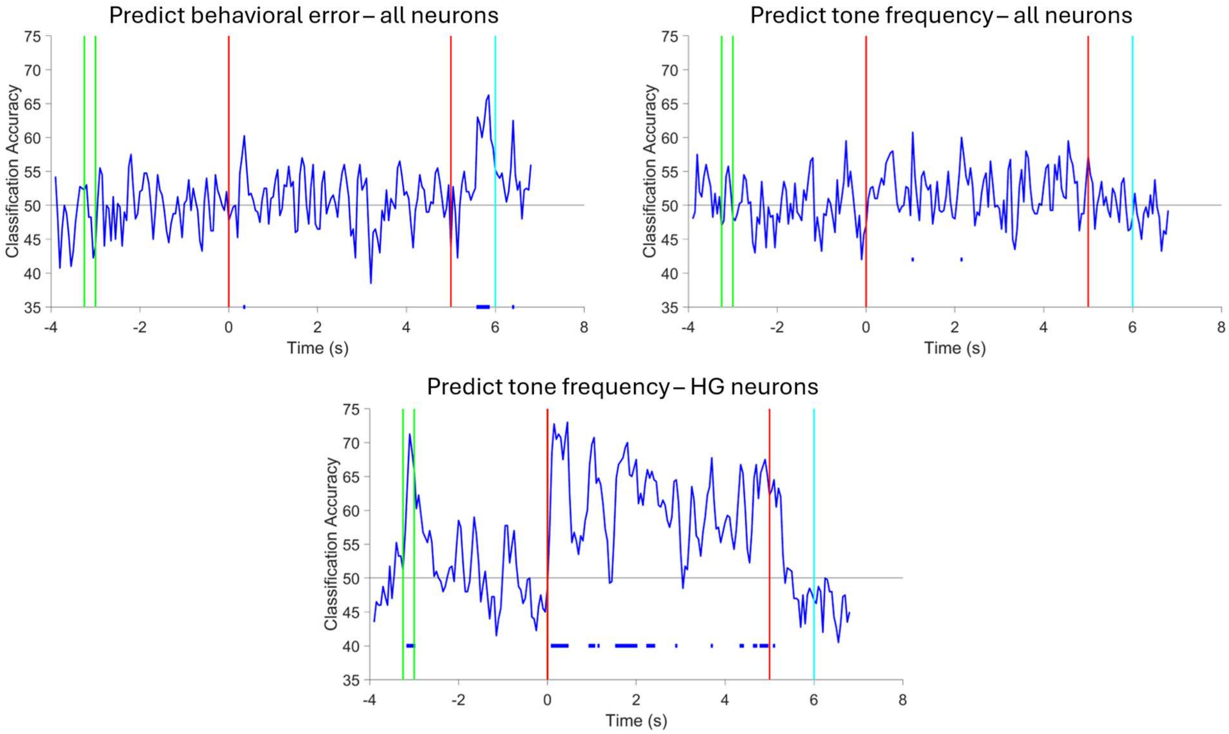

Supplement: 1 [file NIHPP2025.11.10.687666v1-supplement-1.pdf]
